# Benign breast disease and changes in mammographic breast density

**DOI:** 10.1186/s13058-021-01426-7

**Published:** 2021-04-26

**Authors:** Laura L. Reimers, Mandy Goldberg, Parisa Tehranifar, Karin B. Michels, Barbara A. Cohn, Julie D. Flom, Ying Wei, Piera Cirillo, Mary Beth Terry

**Affiliations:** 1 Department of Epidemiology, Columbia University Mailman School of Public Health, New York, NY USA; 2 Herbert Irving Comprehensive Cancer Center, Columbia University Irving Medical Center, New York, NY USA; 3 Department of Epidemiology, Fielding School of Public Health, University of California, Los Angeles, Los Angeles, CA USA; 4 Institute for Prevention and Cancer Epidemiology, Faculty of Medicine and Medical Center, University of Freiburg, Freiburg, Germany; 5 Child Health and Development Studies, Public Health Institute, Berkeley, CA USA; 6 Department of Biostatistics, Columbia University Mailman School of Public Health, New York, NY USA; 7 The Imprints Center for Genetic and Environmental Lifecourse Studies, Columbia University Mailman School of Public Health and the New York State Psychiatric Institute, New York, NY USA

**Keywords:** Mammographic breast density, Benign breast disease, Breast cancer

## Abstract

**Background:**

Mammographic breast density (MBD) and benign breast disease (BBD) are two of the strongest risk factors for breast cancer. Understanding trends in MBD by age and parity in women with BBD is essential to the clinical management and prevention of breast cancer.

**Methods:**

Using data from the Early Determinants of Mammographic Density (EDMD) study, a prospective follow-up study of women born in 1959–1967, we evaluated MBD in 676 women. We used linear regression with generalized estimating equations to examine associations between self-reported BBD and MBD (percent density, dense area, and non-dense area), assessed through a computer-assisted method.

**Results:**

A prior BBD diagnosis (median age at diagnosis 32 years) was reported by 18% of our cohort. The median time from BBD diagnosis to first available study mammogram was 9.4 years (range 1.1–27.6 years). Women with BBD had a 3.44% higher percent MBD (standard error (SE) = 1.56, *p*-value = 0.03) on their first available mammogram than women without BBD. Compared with parous women without BBD, nulliparous women with BBD and women with a BBD diagnosis prior to first birth had 7–8% higher percent MBD (*β* = 7.25, SE = 2.43, *p*-value< 0.01 and β = 7.84, SE = 2.98, *p*-value = 0.01, respectively), while there was no difference in MBD in women with a BBD diagnosis after the first birth (*β* = −0.22, SE = 2.40, *p*-value = 0.93).

**Conclusion:**

Women with self-reported BBD had higher mammographic breast density than women without BBD; the association was limited to women with BBD diagnosed before their first birth.

**Supplementary Information:**

The online version contains supplementary material available at 10.1186/s13058-021-01426-7.

## Background

Mammographic breast density (MBD) is one of the strongest risk factors for breast cancer. Women with 75% or greater mammographically dense tissue have a 4–6-fold risk of breast cancer compared with women with 25% or less dense tissue [1–3]. Benign breast disease (BBD) also increases breast cancer risk 1.5–4-fold depending on histologic subtype, with the highest risk observed in individuals with atypical hyperplasia [4, 5]. Research suggests a link between histologic features, such as intraductal epithelial hyperplasia and lobular microcalcification, and mammographic measures of the breast [6, 7]. Given the importance of these two risk factors, it is essential to understand the relation between the two for improved clinical management and clinical risk assessment.

At least ten studies have examined BBD and MBD, modeling MBD as the outcome (reviewed in [2]). Several studies also examined the effect of MBD on risk of BBD and have reported positive associations between the two [8–10]. The clinical implications from these studies are limited for several reasons. First, most studies used qualitative categorizations of MBD, such as Wolfe classification and/or BI-RADS density categories, which represent broad categories of MBD [2]. For clinical risk assessment and serial assessments over time, more modest changes on the continuous scale of MBD may be important for determining appropriate risk strategies. Second, it is important to understand the directionality of the association between BBD and MBD, but the cross-sectional design of most studies to date cannot provide evidence of causality in either direction. In addition, given that MBD decreases with increasing age [1, 11–14], changes in MBD over time, specifically in women with BBD, could be clinically important to their management and prevention of breast cancer. However, there is a lack of data measuring MBD over time in women with BBD compared with women without BBD. Using a longitudinal birth cohort we evaluated whether self-reported BBD was associated with MBD. Furthermore, since parity induces significant changes in the breast tissue, we further investigated whether any associations between BBD and MBD differed by parity and timing of first birth in relation to BBD diagnosis.

## Methods

We used the Early Determinants of Mammographic Density (EDMD) study, a prospective follow-up of two birth cohorts: the Child Health and Development Study (CHDS) and the Collaborative Perinatal Project (CPP) (previously described in [15–17]), to examine the association of self-reported BBD with MBD and changes in MBD over time. Briefly, women were enrolled in the CHDS in California from 1960 to 1967. We used two sites from the CPP (Boston and Providence) which was conducted from 1959 to 1966. These sites were selected as existing records to conduct active follow-up for the offspring were available (for additional details, see [15–17]). Each cohort recruited pregnant women receiving prenatal care at one of the participating hospitals and followed up their offspring through delivery, birth, and childhood. We successfully traced and enrolled 1134 of female offspring from 2004 to 2008 (see Supplemental Figure 1 for a schematic of the EDMD study design) [15]. We collected data on personal health history, first degree family history of cancer, sociodemographic factors, detailed reproductive history, anthropometric measures, and other lifestyle factors through a 45-min computer-assisted telephone interview with EDMD participants as adults.

In this study, we used data from the adult interviews and MBD data from mammographic films. The interviews included data on race/ethnicity, family history of breast cancer, menopausal status, age at menarche (reported in increments of 0.5 years), and parity. In parous women, we assessed age at first birth, age at last birth, and breastfeeding (ever/never). We calculated body mass index (BMI) in the 20s, 30s, and at interview based on height and weight data reported by participants during the interview. We collected benign breast disease (BBD) diagnosis through self-report on the adult interview. We asked women, “Has a health care provider ever told you that you have fibrocystic breast disease? Sometimes this is also called benign breast disease or multiple cysts in breast”. We then asked women who answered yes at what age they first saw a doctor about their BBD. For our analysis, we excluded 13 women who lacked self-reported BBD information.

We asked participants if they had a mammogram in the 2 years prior to the interview or if they were planning to have one in the following 12 months. From women who had at least one mammogram, we collected signed medical release authorization forms allowing us to request their mammograms for MBD assessment. Mammograms did not follow a set screening schedule for all women and therefore timing of mammograms and time between mammograms varied for each woman. We collected film mammograms from 700 women and assessed breast density using a computer-assisted thresholding program [18]. We calculated absolute breast area and dense area by converting the measure from pixels to cm^2^. We then calculated percent mammographic density by dividing dense area by breast area and multiplying by 100. We calculated non-dense area in cm^2^ as total breast area minus dense breast area. We read MBD from mammogram films in batches of 50 films with repeated readings for 10% of films from the same batch and repeated readings for 10% of films that were included in every batch to assess batch-to-batch variability [15]. We read the left cranio-caudal (CC) film except in cases where the left CC image was unavailable (1% of all films), then we read the right CC film. The within-batch correlation coefficient was 0.96 and the intraclass correlation coefficient was 0.95 [15].

### Statistical analysis

In the 700 women with film mammograms, 691 had available data on the presence/absence of self-reported BBD. We assessed the effects of self-reported BBD (exposure) on MBD (outcome). We excluded 15 women whose only available mammograms were either prior to or at the same age as their BBD diagnosis. Thus, for this analysis, we have 676 women: 119 with a history of self-reported BBD and 557 with no history of self-reported BBD. We had repeated MBD measures on 403 women (59.6%) who had at least one other mammogram measured at least 1 year after the first mammogram.

We compared breast cancer risk factors between those with and without a history of self-reported BBD. We also examined the distribution of each MBD measure by history of self-reported BBD using boxplots. We assessed the association between history of self-reported BBD and MBD using linear regression with generalized estimating equation (GEE) to account for correlation between sibling sets. We also examined the association between age at self-reported BBD diagnosis and MBD by centering the participants’ ages at self-reported BBD diagnosis and including an indicator variable for women without self-reported BBD. Under this parameterization, the coefficient associated with the binary indicator measures the MBD difference between women without self-reported BBD compared to women with self-reported BBD at the mean age and the centered BBD age at diagnosis coefficient represents the effect of an additional year of diagnosis age compared to the mean age at diagnosis. For each woman, we assessed MBD for the first available mammogram. We also assessed MBD for the last available mammogram and calculated the change in MBD from first to last available mammogram for women with repeated measures. To account for differences in the time interval between first and last mammogram between women, we calculated change in MBD per year (i.e., MBD at last mammogram minus MBD at first mammogram divided by age at last mammogram minus age at first mammogram). We examined change in MBD models with and without adjustment for MBD at the first mammogram. In addition, to assess the influence of parity on the relationship between self-reported BBD and MBD, we categorized women into five groups according to parity and self-reported BBD: nulliparous with no BBD, parous with no BBD, nulliparous with BBD, parous with BBD diagnosed prior to first live birth, and parous with BBD diagnosed after first live birth. We excluded two women from these models whose age at BBD was the same as the age at first birth. We adjusted all models for age at mammogram (using age at first mammogram for change in MBD models), race/ethnicity, and BMI in the 30s. This time period of BMI was selected as the median age at self-reported BBD diagnosis in the cohort was 32 years. Lastly, we additionally adjusted models of self-reported BBD, parity, and MBD for family history of breast cancer. We performed analyses using SAS version 9.4 (Cary, NC).

## Results

Table 1 shows the demographic characteristics of our population by history of self-reported BBD. The median age of self-reported BBD diagnosis was 32 years, with a range from 12 to 44 years. The median time between self-reported BBD diagnosis and a woman’s first mammogram was 9.4 years. The median age at first mammogram was 41.3 years and median time between first and last mammograms was 3.3 years in women with self-reported BBD, and 40.8 years and 3.7 years, respectively, in women without self-reported BBD. Average BMI in the 30s was 23.5 ± 5.3 in women with self-reported BBD and 25.0 ± 5.6 in women without self-reported BBD. In addition, 31.6% of women with self-reported BBD and 19.8% of women without self-reported BBD were nulliparous. Boxplots of mammographic breast density at first mammogram (Supplemental Figure 2a-c), last mammogram (Supplemental Figure 3a-c), and change in mammographic density (Supplemental Figure 4a-c), by history of self-reported BBD, are provided in the supplement. In addition, we further explored the relationship between time from self-reported BBD diagnosis to first mammogram and MBD measured at the first mammogram and found no correlation (data not shown).

**Table 1:** Distribution of demographics in women with a history of benign breast disease and no history of benign breast disease, EDMD

Characteristic	BBD (***n*** = 119) ***N*** (%)	No BBD (***n*** = 557) ***N*** (%)
Age at interview, median (range)	44.3 (40.2–47.7)	44.2 (39.2–49.2)
Age at BBD diagnosis, median (range)	32.0 (12.0–44.0)	N/A
Time between BBD diagnosis and first mammogram in years, median (range)	9.4 (1.1–27.6)	N/A
Age at first mammogram, median (range)	41.3 (30.1–46.7)	40.8 (24.7–47.6)
Time between first and last mammogram in years, median (range)	3.3 (1.0–16.6)	3.7 (1.0–19.8)
Race/ethnicity		
Non-Hispanic White	92 (77.3%)	435 (78.4%)
Non-Hispanic African American	12 (10.1%)	72 (13.0%)
Hispanic	9 (7.6%)	28 (5.1%)
Other	6 (5.0%)	20 (3.6%)
Family history of breast cancer		
Yes	18 (15.1%)	70 (12.6%)
No	101 (84.9%)	487 (87.4%)
Menopausal status at interview		
Premenopausal	81 (69.2%)	379 (69.0%)
Perimenopausal	21 (18.0%)	96 (17.5%)
Postmenopausal	15 (12.8%)	74 (13.5%)
BMI in 30s (mean ± std)	23.5 ± 5.3	25.0 ± 5.6
Age at menarche (mean ± std)	13.0 ± 1.7	12.7 ± 1.6
Parity		
Nulliparous	37 (31.6%)	110 (19.8%)
Parous	80 (68.4%)	446 (80.2%)
*Among parous women only:*		
Age at first birth (mean ± std)	26.9 ± 5.3	27.6 ± 5.7
Age at last birth (mean ± std)	31.0 ± 5.7	31.9 ± 5.5
Breastfeeding		
Never	16 (20.0%)	101 (22.7%)
Ever	64 (80.0%)	344 (77.3%)

After adjusting for age, race/ethnicity, and BMI, women with self-reported BBD had on average 3.4% higher percent density on their first available mammogram than women without self-reported BBD (*β* = 3.44, SE = 1.56, *p* = 0.03) (Table 2). The association was similar for percent density at the last available mammogram. Women with self-reported BBD had larger dense area and smaller non-dense area, although these differences were not statistically significant for either the first or the last mammogram.

**Table 2:** Associations between BBD and mammographic breast density (percent MBD, dense area, non-dense area), EDMD

	Adjusted for age, race, and BMI				
Variables	***N***	Estimate	Standard error	***p***-value	95% CI
Percent density, First available mammogram					
BBD	119	3.44	1.56	0.03	0.40, 6.49
No BBD	549	Ref	–	–	–
Percent density, Last available mammogram*					
BBD	67	3.89	2.13	0.07	−0.29, 8.06
No BBD	330	Ref	–	–	–
Dense area, First available mammogram					
BBD	119	2.95	2.14	0.17	−1.24, 7.15
No BBD	549	Ref	–	–	–
Dense area, Last available mammogram*					
BBD	67	2.42	2.86	0.40	−3.19, 8.03
No BBD	330	Ref	–	–	–
Non-dense area, First available mammogram					
BBD	119	−5.94	5.08	0.24	−15.89, 4.01
No BBD	549	Ref	–	–	–
Non-dense area, Last available mammogram*					
BBD	67	−6.59	7.75	0.39	−21.77, 8.59
No BBD	330	Ref	–	–	–

*Only among women with at least 2 available mammograms

Nulliparous women with self-reported BBD and parous women with self-reported BBD prior to first birth had approximately 7–8% higher percent density on first mammogram compared with parous women without self-reported BBD (*β* = 7.25, SE = 2.43, *p* = < 0.01 and *β* = 7.84, SE = 2.98, *p* = 0.01, respectively, adjusted for age, race/ethnicity, and BMI) (Table 3). We also observed higher percent density in women with self-reported BBD prior to first birth on the last available mammogram (*β* = 10.61, SE = 3.80, *p* = 0.01). This suggests that percent density remains high over time in women with self-reported BBD prior to first birth.

**Table 3:** Associations between BBD, parity, and timing of first birth relative to BBD diagnosis and mammographic breast density (percent MBD, dense area, and non-dense area), EDMD

		Adjusted for Age, Race, and BMI			
Variables	***N***	Estimate	Standard error	***p***-value	95% CI
Percent density, first available mammogram					
No BBD, nulliparous	108	3.07	1.79	0.09	−0.44, 6.58
No BBD, parous	440	Ref	–	–	–
BBD, nulliparous	37	7.25	2.43	< 0.01	2.50, 12.01
BBD before first birth	25	7.84	2.98	0.01	2.01, 13.68
BBD after first birth	53	−0.22	2.40	0.93	−4.92, 4.49
Percent density, last available mammogram*					
No BBD, nulliparous	68	2.76	1.88	0.14	−0.92, 6.45
No BBD, parous	262	Ref	–	–	–
BBD, nulliparous	15	4.44	3.94	0.26	−3.28, 12.16
BBD before first birth	19	10.61	3.80	0.01	3.17, 18.05
BBD after first birth	31	0.56	3.13	0.86	−5.57, 6.70
Dense area, first available mammogram					
No BBD, nulliparous	108	−1.77	2.36	0.45	−6.40, 2.86
No BBD, parous	440	Ref	–	–	–
BBD, nulliparous	37	8.26	3.80	0.03	0.82, 15.70
BBD before first birth	25	−0.16	3.53	0.96	−7.09, 6.76
BBD after first birth	53	0.19	3.18	0.95	−6.04, 6.41
Dense area, last available mammogram*					
No BBD, nulliparous	68	1.53	2.96	0.61	−4.27, 7.33
No BBD, parous	262	Ref	–	–	–
BBD, nulliparous	15	6.11	5.50	0.27	−4.66, 16.88
BBD before first birth	19	1.43	3.79	0.71	−6.00, 8.85
BBD after first birth	31	2.12	4.74	0.66	−7.18, 11.41
Non-dense area, first available mammogram					
No BBD, nulliparous	108	−10.85	6.69	0.11	−23.95, 2.26
No BBD, parous	440	Ref	–	–	–
BBD, nulliparous	37	−13.20	7.98	0.10	−28.83, 2.44
BBD before first birth	25	−17.07	7.44	0.02	−31.66, −2.49
BBD after first birth	53	1.65	8.16	0.84	−14.35, 17.65
Non-dense area, last available mammogram*					
No BBD, nulliparous	68	−4.17	7.42	0.57	−18.70, 10.36
No BBD, parous	262	Ref	–	–	–
BBD, nulliparous	15	−11.27	13.53	0.40	−37.80, 15.25
BBD before first birth	19	−20.95	7.10	< 0.01	−34.87, −7.04
BBD after first birth	31	5.93	13.61	0.66	−20.74, 32.60

*Only among women with at least 2 available mammograms

Nulliparous women with self-reported BBD had larger dense area (*β* = 8.26, SE = 3.80, *p* = 0.03) and smaller non-dense area (*β* = −13.20, SE = 7.98, *p* = 0.10) on the first mammogram compared with parous women without self-reported BBD, while women with self-reported BBD prior to first birth had smaller non-dense area (*β* = −17.07, SE = 7.44, *p* = 0.02), but did not differ in amount of dense area (Table 3). Associations between self-reported BBD, parity, and MBD were similar in models additionally adjusted for family history of breast cancer (data not shown).

History of BBD was not related to changes in percent density or dense area, but was positively associated with changes in non-dense area over time (*β* = 1.94, SE = 0.95, *p* = 0.04; Table 4). Women with self-reported BBD after first birth did not differ in terms of percent density or amounts of dense or non-dense area compared with parous women without self-reported BBD, but had increased change in non-dense area over time (*β* = 3.48, SE = 1.59, *p* = 0.03; Table 4). Age at self-reported BBD diagnosis was not associated with measures of MBD at either time point or change in MBD (Supplemental Table 1).

**Table 4:** Associations between BBD with and without considering parity and timing of first birth relative to BBD diagnosis and changes in mammographic breast density (percent MBD, dense area, non-dense area), EDMD

	Adjusted for age^**a**^, race, and BMI					Adjusted for age^**a**^, race, BMI, and MBD at first mammogram			
Variables	***N***	Estimate	Standard error	***p***-value	95% CI	Estimate	Standard error	***p***-value	95% CI
Change in percent density^b^									
BBD	67	−0.21	0.46	0.65	− 1.10, 0.69	− 0.08	0.47	0.86	− 1.00, 0.84
No BBD	330	Ref	–	–	–	Ref	–	–	–
No BBD, nulliparous	68	−0.40	0.39	0.30	−1.16, 0.36	−0.27	0.39	0.48	− 1.03, 0.49
No BBD, parous	262	Ref	–	–	–	Ref	–	–	–
BBD, nulliparous	15	−0.13	1.06	0.90	−2.20, 1.94	0.01	1.07	0.99	−2.09, 2.11
BBD before first birth	19	0.71	0.57	0.22	−0.41, 1.83	1.03	0.59	0.08	−0.12, 2.18
BBD after first birth	31	−1.01	0.67	0.13	−2.32, 0.29	−0.95	0.68	0.16	−2.28, 0.38
Change in dense area^b^									
BBD	67	−0.46	0.63	0.47	−1.70, 0.78	−0.33	0.63	0.61	−1.57, 0.91
No BBD	330	Ref	–	–	–	Ref	–	–	–
No BBD, nulliparous	68	−0.21	0.46	0.65	−1.10, 0.69	−0.13	0.45	0.77	−1.01, 0.75
No BBD, parous	262	Ref	–	–	–	Ref	–	–	–
BBD, nulliparous	15	−0.33	1.32	0.80	−2.92, 2.25	−0.07	1.34	0.96	−2.70, 2.57
BBD before first birth	19	0.38	0.61	0.53	−0.82, 1.58	0.48	0.56	0.39	−0.62, 1.58
BBD after first birth	31	−1.45	1.05	0.17	−3.51, 0.60	−1.30	1.06	0.22	−3.37, 0.77
Change in non-dense area^b^									
BBD	67	1.94	0.95	0.04	0.08, 3.80	1.71	1.02	0.09	−0.29, 3.71
No BBD	330	Ref	–	–	–	Ref	–	–	–
No BBD, nulliparous	68	1.08	1.15	0.35	−1.17, 3.33	0.92	1.09	0.40	−1.23, 3.07
No BBD, parous	262	Ref	–	–	–	Ref	–	–	–
BBD, nulliparous	15	2.21	2.00	0.27	−1.72, 6.13	1.83	2.09	0.38	−2.27, 5.93
BBD before first birth	19	−0.29	0.69	0.68	−1.64, 1.06	− 0.68	0.68	0.31	−2.01, 0.65
BBD after first birth	31	3.48	1.59	0.03	0.37, 6.59	3.43	1.71	0.05	0.08, 6.79

^a^Age at first mammogram

^b^Change in MBD was calculated as MBD at last mammogram minus MBD at first mammogram divided by age at last mammogram minus age at first mammogram

## Discussion

Overall, women with self-reported BBD had higher percent density than women without self-reported BBD. When we further considered BBD diagnosis jointly with parity and age at first birth, we found that nulliparous women with BBD and women with BBD prior to first birth, but not women with BBD diagnosed after first birth, had higher percent density compared with parous women without BBD. While percent density decreases on average over time, our findings suggest that percent density remains high in women with self-reported BBD diagnosed prior to first birth.

Our data support an association between histologic changes in the breast tissue (for which BBD diagnosis is a proxy) and percent MBD, which appears consistent with a positive association seen in previous studies [8, 10]. However, in these prior studies, researchers measured mammographic density prior to BBD diagnosis and, thus, were limited in drawing conclusions about the effect of BBD on mammographic density. In addition, these studies used mammograms collected during the 1980s and estimates of density were subjectively measured by visual inspection into few broad categories [8, 10], whereas our results used a computer-assisted method and continuous measures of different components of density (dense and non-dense areas) as well as percent density.

Age is a key factor that affects mammographic measures [1, 11–14]. We did not observe differences in MBD by age at self-reported BBD diagnosis. Earlier age at BBD diagnosis was associated with greater breast cancer risk among women diagnosed with BBD at the Mayo Clinic from 1967 to 1991 [19]. However, the age at diagnosis ranged from < 30 to ≥70 (mean = 51 years), and the relative risk for breast cancer was similar in women diagnosed at < 30 and 30–39 years. Most women in our cohort, who were age 39–49 years at interview, were diagnosed with self-reported BBD prior to 40 years of age, which may explain why we did not observe an association between age at BBD and density.

Nulliparity and older age at first birth are known breast cancer risk factors [20]. In women without BBD, we observed higher percent density in nulliparous compared with parous women, which is consistent with studies associating parity with lower mammographic density (reviewed in [2, 21]). Women with self-reported BBD were more often nulliparous than women without self-reported BBD in our population. Nulliparous women with self-reported BBD also had higher percent density compared with parous women without self-reported BBD, and the magnitude of association was approximately double what we observed for nulliparous women without BBD (7% vs 3%, respectively). Women with self-reported BBD diagnosed after first birth had similar percent density to parous women without self-reported BBD. However, percent density was higher in parous women with BBD diagnosed prior to first birth, compared to parous women never diagnosed with BBD suggesting that there is not a protective effect of parity on density in women who have already been diagnosed with BBD prior to first birth. Women with BBD prior to first birth had a later age at first birth, which may explain why we did not observe a protective effect of parity in these women, though previous studies examining age at first birth and density have not consistently observed an association between the two (recently reviewed in [22]).

The relation between histological and mammographic measures of the breast has been based on the fact that these changes (e.g., calcifications, adenoma) in tissue correspond to variations in mammographic measures [2, 21]. Previous studies have linked mammographic density with proliferation and appearance of epithelial or stroma tissue (reviewed in [2, 21]). A biological hypothesis posits that genetic and environmental factors affect the proliferative activity and quantity of stromal and epithelial tissue in the breast and that mammographic measures reflect differences [21]. The dense area of the mammogram shows stromal and epithelial tissue, whereas non-dense area shows the fat. Mammographically dense tissue may reflect the proliferation of the breast epithelium and stroma in response to a number of factors [2]. Our data showing higher percent density and higher dense area for women with self-reported BBD supports this, but also suggests that BBD may affect the amount of non-dense area, particularly in women with self-reported BBD prior to first birth. Absolute dense area and percent density are both positively associated with breast cancer risk, while the amount of non-dense area has been inversely associated with risk [23]. Non-dense area and percent density are more strongly correlated with BMI than dense area [24]. The association between self-reported BBD and these measures may reflect differences in body size and fat tissue in the breast in addition to differences in fibroglandular tissue captured by dense area. Non-dense area and percent density are positively and inversely associated with lobular involution, respectively [25], suggesting that less fat tissue in women with self-reported BBD may reflect a smaller degree of lobular involution in the breast. Furthermore, a quantitative study measuring lobular involution in women with both a mammogram and breast biopsy show that mammographic density correlates with amounts of at-risk epithelium [26], suggesting that these measures are markers of breast cancer risk, particularly among premenopausal women. Lobular involution has consistently been shown to be lower among parous women [27], which may be reflected in our population of women with self-reported BBD prior to first birth. Additional data on the histology of BBD in these women could provide additional information on the association between histological changes and mammographic measures. For example, if we were able to stratify this data by atypical hyperplasia versus non-proliferative disease, we may see a stronger association between BBD in those women who had a BBD diagnosis of atypical hyperplasia, as been seen with other studies stratifying by BBD subtype [28–30].

Our ability to use computer-assisted mammographic density measures in women with BBD proved a key strength of this study. In addition, our longitudinal data allowed us to assess change in mammographic density over time, and examine the associations between BBD diagnosed prior to measurement of MBD at different points in time. There are also limitations to our study. First, the BBD diagnosis was based on self-reported data of physician diagnosed BBD. However, the prevalence of women with a history of physician diagnosed BBD in our study (17.6%) is similar to other cohort studies, such as the Women’s Health Study and the California Teachers Study [31, 32]. Self-reported data does limit our analyses as it does not account for the subtype of BBD. BBD is a heterogeneous disease; by lumping all women into one group (e.g.*,* BBD), we potentially mask stronger effects in the more severe cases of BBD. Future research should replicate this study in another BBD population with data on BBD subtype. In addition, our data is limited in the ability to account for weight change between mammograms since we do not have measures of BMI at time of mammograms. We adjusted for BMI in the 30s as the median age of BBD diagnosis was 32 years. We also conducted a sensitivity analysis to assess the change in BMI from the 30s to BMI at interview and observed a similar mean change in BMI over time between women with and without BBD (data not shown). Finally, several different analyses of associations between BBD and different mammographic measures were conducted; these analyses were not adjusted for the multiple comparisons conducted.

## Conclusion

Our results suggest a temporal association between self-reported BBD and higher percent mammographic density, particularly in women who have self-reported BBD diagnosed prior to first birth or are nulliparous. After diagnosis of BBD, women should be followed up either by their primary care physician or by a breast surgeon. One of the important factors of long-term care for these women is accurate risk assessment to guide decisions about screening and prevention. This suggests that risk assessment models incorporating both age at BBD, particularly relative to parity, and MBD should also consider the timing of diagnosis and measurement to accurately assess risk and better target interventions for risk reduction and screening.

## Supplementary Information

**Additional file 1: Supplemental Table 1.** Associations between age at BBD diagnosis and mammographic breast density (percent MBD, dense area and non-dense area), EDMD. **Supplemental Figure 1.** Early Determinants of Mammographic Density (EDMD) study design schematic. **Supplemental Figure 2a-c.** Boxplots of mammographic breast density (percent MBD, dense area and non-dense area) at first mammogram by history of BBD, EDMD. **Supplemental Figure 3a-c.** Boxplots of mammographic breast density (percent MBD, dense area and non-dense area) at last mammogram by history of BBD, EDMD. **Supplemental Figure 4a-c.** Boxplots of change in mammographic breast density between first and last mammogram (percent MBD, dense area and non-dense area) by history of BBD, EDMD. Change in MBD was calculated as MBD at last mammogram minus MBD at first mammogram divided by age at last mammogram minus age at first mammogram.

## Data Availability

The data used and analyzed in this study are available upon request from the corresponding author, Dr. Mary Beth Terry. Requests should be e-mailed to mt146@cumc.columbia.edu.

## Abbreviations

MBD: Mammographic breast density
BBD: Benign breast disease
EDMD: Early Determinants of Mammographic Density Study
CHDS: Child Health and Development Study
CPP: Collaborative Perinatal Project
CC: Cranio-caudal
GEE: Generalized estimating equation
BMI: Body mass index
SE: Standard error
CI: Confidence interval
