# Supplementary material for: Benign breast disease and changes in mammographic breast density
Source: Breast Cancer Res. 2021 Apr 26;23:49. doi: 10.1186/s13058-021-01426-7 (PMC8074418; doi:10.1186/s13058-021-01426-7)
Supplement: Supplementary file 1 — Additional file 1: Supplemental Table 1. Associations between age at BBD diagnosis and mammographic breast density (percent MBD, dense area and non-dense area), EDMD. Supplemental Figure 1. Early Determinants of Mammographic Density (EDMD) study design schematic. Supplemental Figure 2a-c. Boxplots of mammographic breast density (percent MBD, dense area and non-dense area) at first mammogram by history of BBD, EDMD. Supplemental Figure 3a-c. Boxplots of mammographic breast density (percent MBD, dense area and non-dense area) at last mammogram by history of BBD, EDMD. Supplemental Figure 4a-c. Boxplots of change in mammographic breast density between first and last mammogram (percent MBD, dense area and non-dense area) by history of BBD, EDMD. Change in MBD was calculated as MBD at last mammogram minus MBD at first mammogram divided by age at last mammogram minus age at first mammogram. [file 13058_2021_1426_MOESM1_ESM.docx]

**Supplemental Table 1.** Associations between age at BBD diagnosis and mammographic breast density (percent MBD, dense area and non-dense area), EDMD

|  | **Adjusted for Age, Race, and BMI** | | | |
| --- | --- | --- | --- | --- |
| **Variables** | **Estimate** | **Standard Error** | **p-value** | **95% CI** |
| Percent density, First available mammogram |  |  |  |  |
| No BBD | -3.44 | 1.55 | 0.03 | -6.49, -0.40 |
| Age at BBD | 0.00 | 0.19 | 1.00 | -0.36, 0.36 |
|  |  |  |  |  |
| Percent density, Last available mammogram* |  |  |  |  |
| No BBD | -3.94 | 2.15 | 0.07 | -8.15, 0.27 |
| Age at BBD | 0.08 | 0.28 | 0.78 | -0.47, 0.63 |
|  |  |  |  |  |
| Change in percent density*^ |  |  |  |  |
| No BBD | 0.22 | 0.48 | 0.65 | -0.72, 1.15 |
| Age at BBD | -0.01 | 0.05 | 0.84 | -0.11, 0.09 |
|  |  |  |  |  |
| Dense area, First available mammogram |  |  |  |  |
| No BBD | -2.98 | 2.12 | 0.16 | -7.14, 1.19 |
| Age at BBD | 0.33 | 0.33 | 0.33 | -0.32, 0.97 |
|  |  |  |  |  |
| Dense area, Last available mammogram* |  |  |  |  |
| No BBD | -2.88 | 2.90 | 0.32 | -8.57, 2.81 |
| Age at BBD | 0.70 | 0.38 | 0.07 | -0.05, 1.45 |
|  |  |  |  |  |
| Change in dense area*^ |  |  |  |  |
| No BBD | 0.49 | 0.66 | 0.47 | -0.82, 1.79 |
| Age at BBD | -0.04 | 0.08 | 0.64 | -0.19, 0.12 |
|  |  |  |  |  |
| Non-dense area, First available mammogram |  |  |  |  |
| No BBD | 5.89 | 5.06 | 0.24 | -4.02, 15.80 |
| Age at BBD | 0.80 | 0.51 | 0.12 | -0.20, 1.81 |
|  |  |  |  |  |
| Non-dense area, Last available mammogram* |  |  |  |  |
| No BBD | 5.75 | 7.98 | 0.47 | -9.90, 21.39 |
| Age at BBD | 1.27 | 0.83 | 0.13 | -0.35, 2.89 |
|  |  |  |  |  |
| Change in non-dense area*^ |  |  |  |  |
| No BBD | -2.04 | 0.99 | 0.04 | -3.98, -0.10 |
| Age at BBD | 0.14 | 0.11 | 0.21 | -0.08, 0.36 |

*Only among women with at least 2 available mammograms

^Change in MBD was calculated as MBD at last mammogram minus MBD at first mammogram divided by age at last mammogram minus age at first mammogram

**Supplemental Figure 1.** Early Determinants of Mammographic Density (EDMD) study design schematic

**
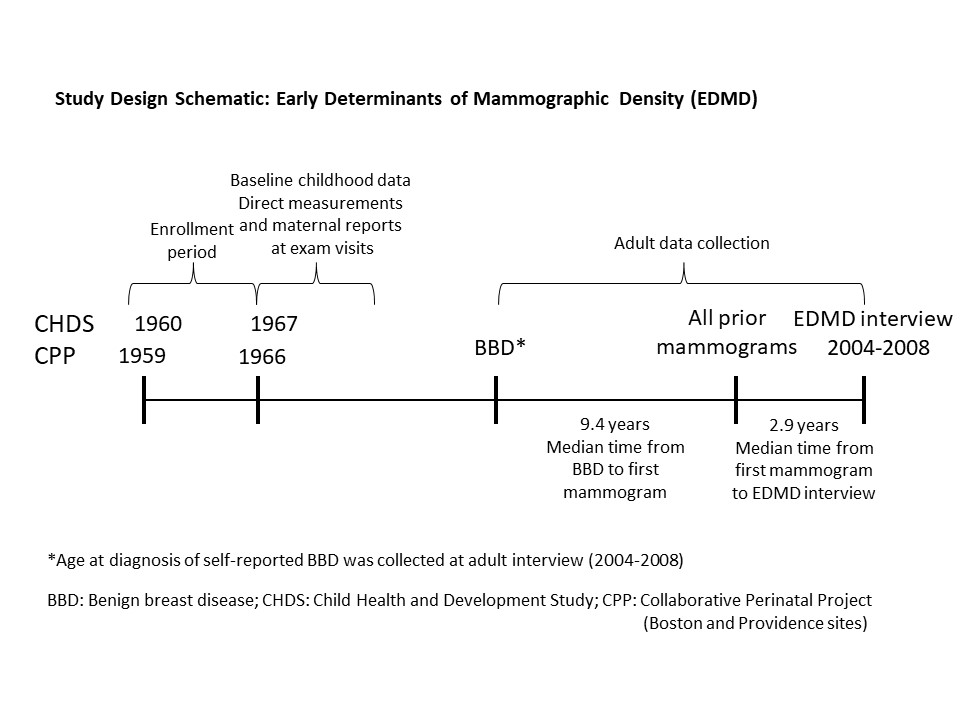
**

**Supplemental Figure 2a-c.** Boxplots of mammographic breast density (percent MBD, dense area and non-dense area) at first mammogram by history of BBD, EDMD

**a**

**b**

**c**

**Supplemental Figure 3a-c.** Boxplots of mammographic breast density (percent MBD, dense area and non-dense area) at last mammogram by history of BBD, EDMD

**a**

**b**

**c**

**Supplemental Figure 4a-c.** Boxplots of change in mammographic breast density between first and last mammogram (percent MBD, dense area and non-dense area) by history of BBD, EDMD. Change in MBD was calculated as MBD at last mammogram minus MBD at first mammogram divided by age at last mammogram minus age at first mammogram

**a**

**b**

**c**
